# Transactions of the Southern Surgical and Gynecological Association

**Published:** 1891-11

**Authors:** 


TRANSACTIONS OF THE SOUTHERN SURGICAL AND GYNECOLOGICAL ASSOCIATION. Vol. III. Third Session, held at Atlanta, Ga., November 11, 12, and 13, 1890. Edited by WM. E. B. DAVIS, M. D., Secretary, and printed by WILLIAM J. DORNAN. Octavo., pp. xli.—444. Philadelphia: Published by the Association. 1891.

The volume before us records the work done by this wonderful Association during its third annual session, held at Atlanta, November 11, 12, and 13, 1890. The organization numbers now about 100 members, and its transactions take rank in scientific value with those of the foremost special societies of the world. It is most superbly edited by the accomplished Secretary of the Association, Dr. W. E. B. Davis, of Birmingham, Ala., and it is printed in Dornan's best style. This is giving the Association great praise, but not more than it merits, for these two men, Davis to edit the volume and Dornan to print it, constitute a team that could not fail to make a good book. The discussions on the papers in this book are very strong, and increase the value of the volume very much. The Association did itself great honor in electing Dr. Lewis S. McMurtry, of Louisville, President for the present year, and he will preside at the coming annual meeting, to be held in Richmond, November 10, 11, and 12, 1891. It is needless to say that Dr. Davis was continued as Secretary, and that these two, President and Secretary, pulling together, will secure an attractive and valuable programme for the approaching meeting. We advise every surgeon and gynecologist, who can do so, to attend, and we further advise all who cannot attend the meeting to purchase the Transactions. They will feel amply repaid for the outlay.

